# Comparative Proteomic Analysis of *Rhizoctonia solani* Isolates Identifies the Differentially Expressed Proteins with Roles in Virulence

**DOI:** 10.3390/jof8040370

**Published:** 2022-04-05

**Authors:** Seenichamy Rathinam Prabhukarthikeyan, Chidambaranathan Parameswaran, Shraddha Bhaskar Sawant, Ramasamy Naveenkumar, Arabinda Mahanty, Umapathy Keerthana, Manoj Kumar Yadav, Annamalai Anandan, Periyasamy Panneerselvam, Manas Kumar Bag, Prakash Chandra Rath

**Affiliations:** ICAR-National Rice Research Institute, Cuttack 753006, India; agriparames07@gmail.com (C.P.); sbsawant56@gmail.com (S.B.S.); naveenrk005@gmail.com (R.N.); arabindamahanty5@gmail.com (A.M.); keerthusrinath@gmail.com (U.K.); m.yadav14@gmail.com (M.K.Y.); anandanau@yahoo.com (A.A.); panneerccri@rediffmail.com (P.P.); manas.bag@gmail.com (M.K.B.); pcrath67@gmail.com (P.C.R.)

**Keywords:** *Rhizoctonia solani* AG1-IA, sheath blight, LC-MS/MS, virulent proteins, fungal proteomics

## Abstract

Sheath blight of rice is a destructive disease that could be calamitous to rice cultivation. The significant objective of this study is to contemplate the proteomic analysis of the high virulent and less virulent isolate of *Rhizoctonia solani* using a quantitative LC-MS/MS-based proteomic approach to identify the differentially expressed proteins promoting higher virulence. Across several rice-growing regions in Odisha, Eastern India, 58 *Rhizoctonia* isolates were obtained. All the isolates varied in their pathogenicity. The isolate RS15 was found to be the most virulent and RS22 was identified as the least virulent. The PCR amplification confirmed that the RS15 and RS22 belonged to the *Rhizoctonia* subgroup of AG1-IA with a specific primer. The proteomic information generated has been deposited in the PRIDE database with PXD023430. The virulent isolate consisted of 48 differentially abundant proteins, out of which 27 proteins had higher abundance, while 21 proteins had lower abundance. The analyzed proteins acquired functionality in fungal development, sporulation, morphology, pathogenicity, detoxification, antifungal activity, essential metabolism and transcriptional activities, protein biosynthesis, glycolysis, phosphorylation and catalytic activities in fungi. A Quantitative Real-Time PCR (qRT-PCR) was used to validate changes in differentially expressed proteins at the mRNA level for selected genes. The abundances of proteins and transcripts were positively correlated. This study provides the role of the proteome in the pathogenicity of *R. solani* AG1-IA in rice and underpins the mechanism behind the pathogen’s virulence in causing sheath blight disease.

## 1. Introduction

*Rhizoctonia solani* Kuhn (Teleomorph: *Thanatephorus cucumeris* (Frank) Donk) is a soil-borne necrotrophic fungal pathogen that affects many food crops that are important to humankind. Fourteen anastomosis groups (AG1–AG13 and AGBI) have been assigned based on hyphal fusion [1]. The rice sheath blight causal organism, *R. solani* subgroup of AG1-IA, leads to severe economic losses to rice growers worldwide. Under favorable conditions, the pathogen could cause significant yield losses, affecting as much as 50% of the entire yield [2]. The severity of the disease depends on the variety, plant age, climatic factors and excessive use of nitrogenous fertilizers. *R. solani* AG1-IA produces necrotic, dark to reddish-brown color, oval or elliptical-shaped lesions on the leaf sheath, blade and culm of rice [3,4]. Unavailability of resistant donors, pathogen variability, prolonged existence as sclerotia in soil and lack of promising crop protection strategies make it challenging to manage sheath blight disease [5].

The *Rhizoctonia* population is diverse, considering its discrete cultural, morphological, pathological and physiological characteristics [6]. *R. solani* AG1-IA isolates are genetically highly variable. Variability is a vital factor that degenerates the static mechanism of host resistance. Different molecular approaches, such as DNA-based sequence homology restriction analysis of ribosomal DNA (RAPD), have confirmed that *R. solani* isolates are genetically more diverse [7,8]. Principal factors affecting the virulence of *R. solani* include cell-wall-degrading enzymes and necrosis-inducing factors. Fungal-secreted proteins, known as effectors, are critical for host cell infection, as they impede their immune system and are consequently responsible for the pathogenicity of the necrotrophs [9]. Whole-genome sequencing of *R. solani* AG1-IA manifests three genes that encode effector proteins, including peptidase inhibitor I9 domain, cytochrome C oxidase assembly protein CtaG/cox11 domain, and glycosyltransferase GT family 2 domain; they are involved in necrosis-inducing activities in rice and maize crop [10]. Nagarajkumar et al. [11] reported the production of higher amounts of oxalic acid by a virulent isolate of *R. solani* AG1-IA than the less virulent isolate. A significant amount of oxalate increases the receptivity of plant tissues to pathogen infection. Recently, the polygalacturonase (AG1IA_04727) gene was substantial in *R. solani* pathogenesis [12]. Hence, the knowledge of the virulence spectrum of *Rhizoctonia* isolates and their discrepancy is crucial for effective disease management strategies, including the development of sheath blight-resistant cultivars through available breeding programs [13] or genetic engineering or fungicides as well as biological control [14,15,16].

Proteomics was proven a significant tool to interpret the molecular mechanism behind biological systems. Proteomics is the study of the whole set of proteins expressed in each given condition in a particular cell. Unlike the static genome, the proteome is highly dynamic. The last two decades provided considerable knowledge on functional information of abiotic and biotic stress responses [17]. Proteomics allows us to accurately predict the expression profile of the final gene product, which is generally not offered by transcriptomics or genomics. Gel-based and gel-free proteomics approaches were used to determine differentially expressed proteins in plant and fungal species [18,19,20,21]. Their comparative proteome analysis could comprehend the pathogenicity, metabolism, infection process, stress response, broad host range, signal transduction and candidate virulence protein identification of fungal species and related strains [22]. Using a two-dimensional polyacrylamide gel electrophoresis analysis (2-DE), Manikandan et al. [20] reported that the *F. oxysporum* f.sp. *lycopersici* virulent isolate (FOL-8) had 14 differentially expressed proteins implicated in pathogenicity, infection and disease development in tomato root tissue. The analysis of 1-DE and 2-DE gel electrophoresis demonstrated about 75 proteins expressed differentially during the sclerotial maturation of *R. solani* AG-1. The identified proteins were involved in the essential fungal metabolism and pathogenicity of *R. solani* [23]. Anderson et al. [24] applied mass spectrometry-based proteomics to identify the pathogenesis-related protein in *R. solani* AG8. *R. solani* AG 4 isolate Rs23A revealed proteins with a possible role in fungal virulence, pathogenicity and cellular processes, as was demonstrated by proteomic profiles of its extracellular and mycelial proteins [25]. Comparative secretome analysis demonstrated that I9 domain-containing proteins were abundant in diverse *R. solani* isolates that can cause plant cell death [26]. Proteomic responses to *R. solani* were investigated using an eight-plexiTRAQ technique (isobaric tags for relative and absolute quantitation) in resistant (Teqing) and susceptible (Lemont) rice cultivars. In total, 755 proteins were differentially expressed, with the majority of them involved in plant metabolism and defense [27].

However, knowledge about differentially expressed proteins promoting virulence between different *R. solani* AG1-IA isolates is limited. Although the molecular basis of *R. solani*-Rice interaction may be well presumed, the mechanism that underpins differences in the virulence factors between various *R. solani* AG1-IA isolates was not conclusive. Protein profiling of virulent and less virulent isolates provides an opportunity to identify the virulence factors. Label-free proteomics has certain advantages, such as reliability, accuracy, fewer experimental errors and a convenient approach in proteomics studies [28,29]. With this background information, the present study is undertaken to identify the differentially expressed proteins among *R. solani* isolates using a quantitative LC-MS/MS-based proteomic approach.

## 2. Materials and Methods

### 2.1. Isolation of Sheath Blight Pathogen

Infected rice plant sheaths and leaves exhibiting typical symptoms of sheath blight disease were gathered across various rice-growing areas of Odisha (40 locations from seven districts), Eastern India, during 2019–2020 and brought to the laboratory to isolate the causal organism. From each location 3–4 rice fields were selected randomly when the crop was at tillering to maturity stage. The specimens were gently rinsed in tap water, cut into small pieces (approximately 1 cm) and then sterilized for 1 min using 1% sodium hypochlorite solution, followed immediately by three sterile distilled water washes and blotted out between sterilized filter papers. The dried tissue bits were placed on a water agar medium and incubated at 27 ± 1 °C for 1–2 days. The emerged hyphal tips were examined under a microscope and then transferred to a Petri dish containing a medium of potato dextrose agar (PDA). The pure cultures in PDA slants were later stored at 4 °C.

### 2.2. Pathogenicity and Disease Assessment

At the ICAR-National Rice Research Institute, Cuttack, India (85°55′48″ E longitudes and 20°26′35″ N latitude), the experiment was implemented. The sheath blight susceptible cultivar, Tapaswini, was used for the experiment. Seeds were sterilized with a 2% sodium hypochlorite solution and washed with sterile distilled water before sowing. Then, seeds were sown in a pot (45 × 60 cm) with autoclaved pot mixture. The 25-day-old rice seedlings were transplanted at two seedlings per hill with three hills per pot (45 × 60 cm). The 58 *R. solani* isolates were grown on PDA medium with seven-day-old mycelial plugs embedded beneath the leaf sheath of 60-day-old plants. The inoculated sheaths were covered with absorbent cotton to maintain humidity. The plants without pathogen inoculation were maintained as healthy controls. The experiment employed a completely randomized block design. Every treatment had three replications with three pots in each and the experiment was repeated twice. Disease progress was documented on the 7th, 14th, 21st and 28th day after pathogen inoculation. The relative lesion height (RLH = lesion height/Plant height × 100) was recorded as described by Sharma et al. [30]. The disease severity was calculated based on the RLH percentage. The data of RLH was converted into a disease index based on a disease score with a 0 to 9 rating scale [31], where 0 represented no infection; 1, lesion was limited to 20%; 3, 21–30%; 5, 31–45%; 7, 46–65%; 9, more than 66–100% of plant height [32]. Based on disease reaction, isolates were categorized into four groups: highly virulent (8–9), moderately virulent (4–7.9), less virulent (1–3.9) and avirulent (0). The virulence index was calculated by averaging the disease score. The percentage disease index (PDI) was calculated following Wheeler [33]. PDI = Sum of all rating/(Total no. of observations × Maximum rating scale) × 100.

### 2.3. Virulence Test on Other Rice Genotypes

The selected highly virulent (RS15) and less virulent (RS22) isolates were inoculated on other genotypes viz., Pusa Basmati-1, Vanaprabha, Hazaridhan, Swarna, Lunishree, Savitri, Sadabahar and TN1. The preparation of inoculums, method of artificial inoculation and disease assessments were similar to those in Section 2.2. The experiment was conducted with three replications in a completely randomized manner. Each replication had five pots with three hills (two seedlings/hill) in each pot. The experiment was repeated twice.

### 2.4. Molecular Characterization

The highly virulent and less virulent isolate of *R. solani* was characterized at the molecular level using ITS and AG1-1A primers. The fungal culture was grown on potato dextrose broth (PDB) for three days at 27 ± 1 °C. The fungal mycelium was harvested and finely ground with liquid nitrogen. The fungal genomic DNA was extracted as per the procedure by the manufacturer (Qiagen fungal DNA extraction kit). A thermal cycler (Eppendorf Master Cycler Gradient, (St. Louis, MO, USA) was used to amplify the ITS by applying the primer pair ITS1:CCTGTGCACCTGTGAGACAC and ITS4: TGTCCAAGTCAATGGACTAT with the following PCR conditions: 94 °C for 5 min; 35 cycles of 94 °C for 1 min; 56 °C for 1 min and 72 °C extension for 1.5 min; and a final extension at 72 °C for 10 min [34]. Furthermore, the AG1-1A specific primers 5′-CTCAAACAGGCATGCTC-3′ and 5′-CAGCAATAGTTGGTGGA-3′ were used to identify *R. solani* as the subgroup of AG1-1A. A total of 25 μL PCR reaction mixture contained 12.5 μL Mix (Bangalore Genei, India), 0.5 μL of forward primer, 0.5 μL of reverse primer, 0.5 μL Taq enzyme, 1 μL of fungal DNA and 10 μL Nanopure water. The PCR reaction was executed with the following program: 94 °C for 4 min; 30 cycles of 94 °C for 1 min and 54 °C for 2 min; 72 °C for 3 min; and the final extension at 72 °C for 7 min [35]. The 25 μL of PCR products were analyzed on 1.5% agarose gel (Sigma-Aldrich, St. Louis, MO, USA), and a gel documentation system (Biorad, Berkley, CA, USA) was used to analyze the ethidium bromide-stained DNA bands. The PCR product was purified using a QIA quick gel extraction kit (Qiagen, Inc., Chatsworth, CA, USA) as per the manufacturer’s instruction. The purified PCR products of ITS and AG1-1A were sent for DNA sequencing at AgriGenome Labs Pvt Ltd., Kochi, India. The DNA sequences were compared with existing *R. solani* sequences available in the GenBank database (www.ncbi.nlm.nih.gov), and submitted to GenBank applying the Bankit sequence submission tool.

### 2.5. Protein Extraction

*R. solani* isolates, RS15 and RS22, were cultured on PDB for three days at 27 ± 1 °C. Sterile filter paper was used to collect the mycelial mat, washed thrice with sterile distilled water and liquid nitrogen was used to obtain a fine powder. The TCA (sigma-76-05-1)-acetone (Merck-100014) method was employed to precipitate the proteins. The samples were brought up to 10% TCA and incubated at −20 °C overnight. The samples were centrifuged for 10 min at 13,000 rpm to obtain an intact pellet and the supernatant was separated. Acetone wash (added 500 μL of acetone to the pellet and incubated for 10 min at −20 °C and again centrifuged for 10 min at 13,000 rpm) of the pellet was performed thrice. Finally, the air-dried pellet was dissolved in a 50 mM ammonium bicarbonate (Fluka analytical FL40867) buffer. The samples were solubilized in 100 µL of 50 mM ammonium bicarbonate (NH_4_HCO_3_) with 1% SDS, mixed well by vortexing followed by 20 min sonication and centrifuged for 10 min at 10,000 rpm. The supernatant was quantified using the Bicinchoninic Acid assay, in which proteins were reduced from Cu^+2^ to Cu^+1^ in an alkaline solution, resulting in the formation of the bicinchoninic acid-induced purple color. The protein samples were incubated along with the standard at 37 °C for 60 min and the spectrophotometer reading was noted at 562 nm. From all the samples, an equal amount of protein was loaded on a 12% 1D SDS-PAGE, silver-stained gels were observed for bands and an Epson Expression 11000XL Scanner was used to scan them.

### 2.6. In Solution Protein Digestion

The extracted protein (100 μg) was digested, treated for 1 h at 95 °C with 100 mM dithiothreitol (DTT), and exposed for 45 min at room temperature in the dark to 250 mM iodoacetamide. With the addition of 4 μg of trypsin, digestion of the protein samples was achieved at 37 °C overnight. The peptides were resuspended in 0.1% formic acid (50 µL) and incubated for 45 min at 37 °C. The supernatants were transferred into a separate tube after centrifugation at 10,000× *g*. The speed vacuum concentrator (Basic Concentraor, Eppendorf, Hamburg, Germany) was used to vacuum dry (45 °C for 6 h at 800 Pa in VAQ mode) the resulting samples and was dissolved in 0.1% formic acid in water (20 μL) for LC-MS/MS.

### 2.7. LC-MS/MS Analysis

An ACQUITY UPLC system (Waters, UK) was used to perform liquid chromatography by injecting 10 μL of the protein samples. The ACQUITY UPLC BEH C18 column (150 mm × 2.1 mm × 1.7 µm) (Waters, UK) separated the samples. Three runs will be performed on each sample to achieve label-free quantification. A gradient elution program was performed for chromatographic separation with mobile phase A (0.1% formic acid in water) and mobile phase B (0.1% formic acid in acetonitrile). Mass spectrometric detection was performed with an electrospray ionization (ESI) source equipped with an SYNAPT G2 QTOF (Waters, UK). Sample analysis was performed in a positive mode.

### 2.8. Peptide Identification and Data Analysis

MassLynx 4.1 WATERS was used to process raw data. The protein identification on PLGS (Protein Lynx Global Server) software 3.0.2, WATERS involved matching MSMS spectra of individual peptides to the database sequence. The sample runs were processed following specific search parameters in the software: Peptide tolerance (ppm): 50; min no. of fragment matches for peptides: 2; fragment tolerance (ppm): 100; min no. of peptide matches for proteins: 2; min no. of fragment matches for proteins: 5; missed cleavages: 1. The sample cysteine sites were modified during processing to carbamidomethylated cysteine, and the methionine sites were considered as a variable modification to the mass, which was prone to oxidation. The Swissprot protein database for *R. solani* was used to search for proteins present in the sample, and a 5% false discovery rate was used. The expression score was normalized using PLGS software, and the mean abundance of a particular protein in two samples was determined by ratio calculation. A *t*-test determined the differences between isolates RS15 and RS22. Protein abundance RS15 to RS22 with at least two-fold differences was considered for up-regulation and 0.5-fold changes for down-regulation. The differentially abundant proteins were classified according to their molecular functions, biological processes and protein class using the Panther online tool (http://www.pantherdb.org/pathway/ (accessed on 10 January2022)).

### 2.9. Quantitative Real-Time PCR Analysis

*R. solani* isolates RS15 and RS22 were cultured on PDB for 3 days at 27 ± 1 °C, and the mycelial mat was collected from three biological replicates and immediately ground with liquid N_2_ for RNA extraction. Three technical replicates per biological replicates were analyzed during qRT-PCR. Total RNA was extracted using the RNeasy Mini kit (QIAGEN, Hilden, Germany). The isolated RNA quality was checked in 1.5% agarose gel and quantified by a NanoDrop^®^ ND-1000 UV-Vis spectrophotometer (Thermo SCIENTIFIC, Walthem, MA, USA). The residual DNA was removed with an RNase-free DNase I enzyme (QIAGEN, Hilden, Germany). According to the manufacturer’s instructions, the complementary DNAs were synthesized from 1 µg of total RNA using the QuantiTect^®^ Reverse Transcription Kit (QIAGEN) in a 20 µL reaction mixture. The qRT-PCR specific primers were designed using the Primer Quest^TM^tool (Integrated DNA Technologies, Coralville, IA, USA) and all primer information is listed in Supplementary Table S1. The RT-PCR was performed on a BIORAD CFX96 Real-Time system, with a total of 10 µL PCR reaction mixture containing 1.0 µL of diluted template cDNA, 5.0 µL of 2× buffer SYBR Green (QIAGEN, Germany), 0.5 µL of each forward and reverse primer and 3.0 µL of sterilized nanopure water. The RT-PCR amplification conditions were as follows: pre-incubation for 95.0 °C for 15 min, and three-step amplification at 94.0 °C for 0.15 s, 60 °C for 0.30 s and 72 °C for 0.30 s and 39 cycles, followed by 95.0 °C for 0.05 s, 65 °C for 0.05 s and 95 °C for 0.5 s. The Rhi-18S rRNA (Forward: ATGATAACTCGACGGATCGC; Reverse: CTTGGATGTGGTAGCCGT) was used as the internal control to normalize the expression of each gene, and the specificity of amplicons was verified by a melting curve analysis using the peak values [36]. Additionally, specificity was confirmed by running a 2.5% agarose gel. Fluorescence was measured at the end of every 72 °C extension phase. The final threshold cycles (Ct) values were the means of three values, including three biological replicates and three technical replicates per biological replicates. The relative expression level of each gene was calculated using the 2^-ΔΔCt^ method. The Ct values for the housekeeping gene was subtracted from the gene of interest to obtain a Ct value. The Ct value of the mock-inoculated control sample was subtracted from the ΔCt value to obtain the ΔΔCt value = Ct Target-Ct Reference. Each fold change in expression level relative to that of the control was expressed as 2^-ΔΔCt^ and all experiments were repeated thrice.

### 2.10. Statistical Analysis

The IRRISTAT v.92-1 program (Biometric Unit, International Rice Research Institute, Los Baños, Laguna, Philippines) was utilized for the data analysis. Data were subject to an analysis of variance (ANOVA, New Providence, NJ, USA), and the data in percentages were arcsine transformed before the analysis. The treatment means were compared by the Duncan’s multiple range test (DMRT) [37].

## 3. Results

### 3.1. Isolation, Pathogenicity and Molecular Characterization

Overall, 58 isolates were identified from various rice-growing areas of Odisha, Eastern India. Artificial inoculation on the rice cultivar Tapaswini demonstrated that typical symptoms of sheath blight disease were produced in all isolates, which displayed differences in virulence, disease index and disease progress. RS15 recorded 100% PDI among the isolates, followed by RS34 (92.59%). The isolates RS16, RS17, RS22, RS26 and RS27 had the lowest PDI of 11.11%. Based on disease reaction, the 58 isolates were categorized into highly virulent (2), moderately virulent (31) and less virulent (25). The virulent index varied from 1 to 9 (Table 1).

**Figure 1:**
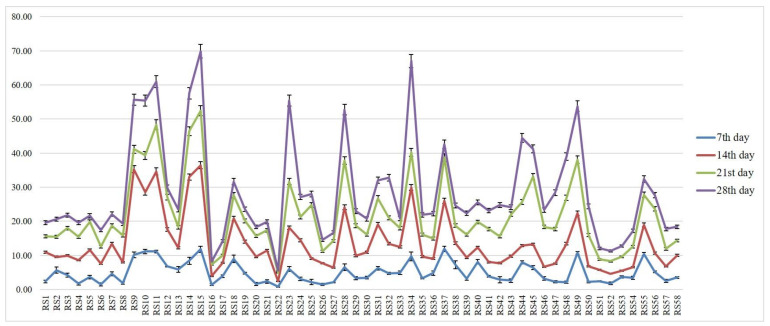
Relative Lesion Length (RLH) % of different *R. solani* isolates on different time intervals. Vertical bars indicate the standard error of three replications. Analysis of variance was performed through DMRT with IRRISTAT.

The RLH was recorded on the 7th, 14th, 21st and 28th days after pathogen inoculation. Among the isolates, RS15 demonstrated the maximum percentage of RLH, and RS22 demonstrated the most negligible percentage of RLH at different time intervals (Figure 1).

Isolates RS15 and RS22 were characterized at the molecular level by sequencing the rDNA internal transcribed spacer region using ITS and AG1-1A specific primers. The DNA sequences were matched with the NCBI GenBank BLAST analysis (http://www.ncbi.nlm.nih.gov (accessed on 5 March 2021)). The resultant sequences were submitted to GenBank, and the accession numbers of MW762960, MW757241 for RS15 and MW757249 and MW757239 for RS22 were obtained, respectively.

### 3.2. Determination of Virulence on Other Rice Genotypes

**Figure 2:**
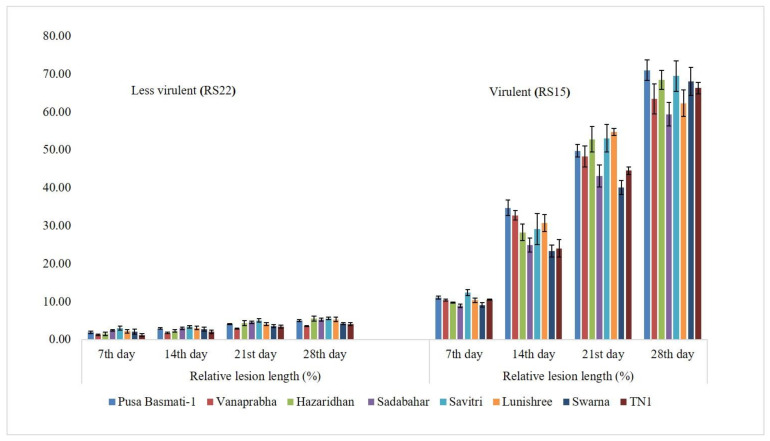
Relative Lesion Length % of RS22 and RS15 on different rice genotypes. Vertical bars indicate the standard error of three replications. Analysis of variance was performed through DMRT with IRRISTAT.

The virulence spectrum of RS15 and RS22 was evaluated on eight rice genotypes *viz*., Pusa Basmati-1, Vanaprabha, Hazaridhan, Swarna, Lunishree, Savitri, Sadabahar and TN1. The plants challenged with RS15 demonstrated higher RLH % on different genotypes than the RS22-inoculated plants on the 7th, 14th, 21st and 28th day, after pathogen inoculation. The disease progress was significantly increased in RS15-inoculated plants, which were low in RS22-inoculated plants (Figure 2).

### 3.3. Proteomic Analysis

A comparative proteomic analysis was performed for virulent (RS15) and less virulent (RS22) isolates using the LC-MS/MS analysis to identify the proteins related to virulence. The results demonstrated that the virulent isolate comprised 48 disparately abundant proteins, out of which 27 proteins had higher abundance and 21 had lower abundance in the virulent isolate. The proteomic information generated has been archived in the PRIDE database and can be accessed with PXD023430 [38,39]. Among the differentially abundant proteins, JmjC domain-containing histone demethylation protein 1, sorting nexin-4, rRNA biogenesis protein RRP36, Exportin-T, Heat shock 70, topoisomerase 1, cyanate hydratase, pyranose 2-oxidase, actin-like protein ARP6, phenylalanine ammonia-lyase and squalene synthase were the most predominant with an increased abundance in the virulent isolate. Similarly, GMP synthase, elongation factor G, glycylpeptide N-tetradecanoyl transferase, sulphate adenylyltransferase, 5′-3′ exoribonuclease 2, glyceraldehyde-3-phosphate dehydrogenase, protein phosphatase methylesterase and protein CFT1 were found to be depleted (Table 2).

### 3.4. Pathway Analysis

**Figure 3:**
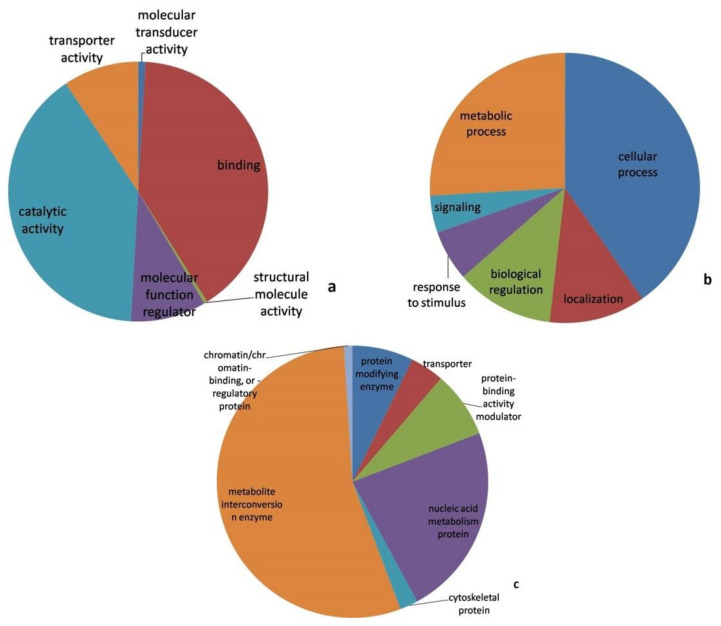
Classification of differentially abundant protein based on (**a**). Molecular functions (**b**). Biological processes (**c**). Protein class as determined by the Panther protein classification tool.

A pathway analysis using the Panther online tool revealed the molecular functions, biological processes and protein class in which differently abundant proteins were involved, as depicted in Figure 3.

Catalytic activity (GO: 0003824) and binding (GO: 0005488) were the most predominantly represented molecular functions. Similarly, the cellular process (GO:0009987), metabolic process (GO: 0008152), biological regulation (GO: 0065007) and localization (GO: 0051179) were the primarily represented biological processes. The predominant protein classes represented by differentially abundant proteins were the metabolite inter-conversion enzyme (PC00262), nucleic acid metabolism protein (PC00171) and protein-binding activity modulator (PC00095).

### 3.5. Validation of Differentially Expressed Proteins by qRT–PCR

**Figure 4:**
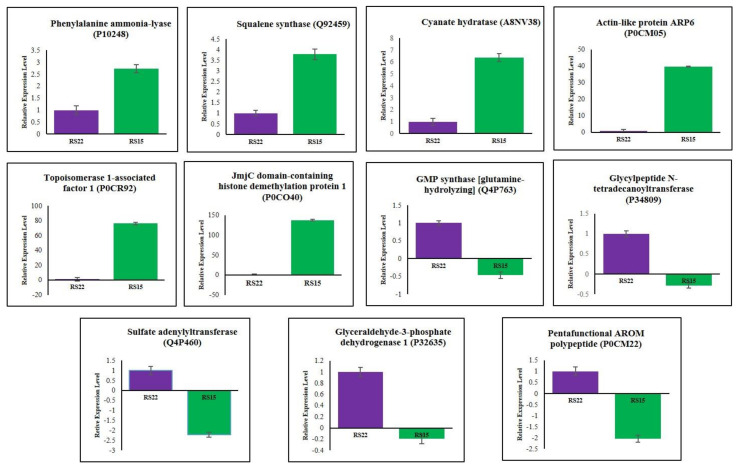
The relative mRNA expression levels of eleven selected genes in virulent (RS15) and less virulent isolate (RS22) of *R. solani*. The mRNA levels of each gene were normalized against expression of Rhi-18S rRNA as a reference gene. Error bars represent standard error from three individual biological replicates.

qRT–PCR was performed to investigate the changes of differentially expressed proteins at mRNA levels in RS15 and RS22. The six up-regulated proteins viz., phenylalanine ammonia-lyase (P10248), squalene synthase (Q92459), cyanate hydratase (A8NV38), actin-like protein ARP6 (P0CM05), topoisomerase 1-associated factor 1 (P0CR92) and JmjC domain-containing histone demethylation protein 1 (P0CO40), and five down-regulated proteins, such as GMP synthase [glutamine-hydrolyzing] (Q4P763), glycylpeptide N-tetradecanoyltransferase (P34809), sulfate adenylyltransferase (Q4P460), glyceraldehyde-3-phosphate dehydrogenase 1 (P32635) and pentafunctional AROM polypeptide (P0CM22), were randomly selected for gene expression analysis. The relative mRNA levels of each transcript were checked in RS15 and RS22. Each gene’s mRNA levels were normalized to the expression of Rhi-18S rRNA as a control gene in each sample. The qRT–PCR results demonstrated a positive correlation of the mRNA expression level with the changes in protein abundance levels for all eleven genes (Figure 4).

The relative fold changes of mRNA and protein level were positively correlated for eleven genes with a 95% correlation (*r* = 0.95) that indicated a strong agreement between the qRT-PCR analysis and the proteomic data. This result confirmed our proteomics data and implied that the proteomics results were reliable.

## 4. Discussion

Across different rice ecosystems in Odisha, eastern India, 58 isolates of *R. solani* were isolated. Koch’s postulates were proven for the sheath blight pathogen on the rice cultivar, Tapaswini. The isolates varied in their virulence from 11.11% to 100%. The virulence diversity among *R. solani* isolates have been well documented by many researchers [13,40,41,42,43]. We have used both universal ITS and species-specific *R. solani* AG1-IA primers to identify at the molecular level. The PCR amplification and sequencing results revealed that RS15 and RS22 belonged to the *R. solani* AG1-IA. These results were supported by Lore et al. [13] and Wang et al. [9], who differentiated *R. solani* isolates using species-specific AG1-IA primers. Similarly, Yu et al. [44] used AG1-IA specific primers and identified that 220 strains of *R. solani* isolates collected from 11 provinces of China belonged to *R. solani* AG1-IA.

Several researchers have well documented the morphological and genetic diversity of *R. solani* isolates [45,46,47]. However, much work has not been published on the fungal proteomics of this specific pathogen. Variations in protein expression levels among the isolates could be studied from the protein profiles of pathogens. It helps identify proteins commonly present within the population of certain pathogens. Fungal proteomics has become a suitable tool to obtain molecular maps of pathogenicity and virulence factors; thus, new insights were opened to detect and protect plant pathogens. Fungal-secreted proteins contribute to virulence and fungal pathogenicity control [20]. In this aspect, virulence-responsive protein identification is most important to uncover the mechanism of the pathogenic process in plants. Hence, we attempted to compare the proteomic profiles of the highly virulent RS15 isolate with the less virulent RS22 isolate using a quantitative LC-MS/MS-based proteomic approach to identify differentially expressed proteins that promote virulence. A total of 27 proteins were differentially expressed with higher abundance in the virulent isolate RS15, of which there existed a greater abundance of protein P0CR62; sorting nexin-4 was highly up-regulated in RS15 compared to RS22. Deng et al. [48] reported that sorting nexin is essential for *Magnaporthe* conidiation and pathogenicity. The Snx41 mutant strain exhibited reduced aerial hyphal growth and defects in conidiation and pathogenicity of *M. oryzae*. Similarly, Zheng et al. [49] reported that sorting nexin MoVps17 null mutation is responsible for defective growth, development and pathogenicity in *M. oryzae*. Our results suggest that higher expression of sorting nexin might support fungal development and virulence of *R. solani* (RS15).

In our study, the proteins P0CO40 and P0CO41 were identified as the histone demethylation protein 1 containing the JmjC domain. These play a vital role in histone demethylation and transcriptional regulation of secondary metabolite gene clusters in *Aspergillus nidulans* and phytopathogenic fungus *Fusarium graminearum* [50,51]. Huh et al. [52] conducted a functional analysis of a putative JmjCdomain-containing histone demethylase in *Magnaporthe oryzae*. The fungal development requires *MoJMJ1-*encoding JmjC histone demethylase, especially in the pre-penetration phase of *M. grisea* [52]. From these supportive findings, our results suggest that the over-expression of P0CO40 and P0CO41 proteins enhanced the pathogenicity of virulent isolates.

The protein P18694 was identified as Heat shock 70 kDa (Hsp70) protein 2, which was overexpressed in RS15. Hsp70 is highly conserved and has a prominent role in the fungal system for growth, morphogenesis and various stress conditions [53,54]. Dnj1 in *Ustilago maydis* is imperative for pathogenicity and was characterized as part of a conserved cellular response to ER stress [55]. Yang et al. [56] revealed that heat shock proteins *MoSsb1*, *MoSsz1* and *MoZuo1* and their functional mechanisms are involved in regulating the conidiation, pathogenicity, vegetative growth and mating of rice blast fungus. Similarly, *Fg*Hsp90 is required for the virulence sexual and asexual development of the plant pathogenic fungus, *Fusarium graminearum* [57]. Chen et al. [54] demonstrated that *FpHsp70* genes were up-regulated when infected with *F*. *pseudograminearum*, implying their significance in the virulence of *F*. *pseudograminearum*. This supporting information suggested an up-regulation of Hsp70 in virulent isolates that plays a significant role in sexual development, sporulation and virulence.

Another up-regulated protein was P0CR92, Topoisomerase 1, which relaxes the supercoiled DNA and enables numerous basic cellular processes, including transcription, replication and recombination [58]. In wheat ear infection assays, *top1* mutants of *F. graminearum* and *F. culmorum* demonstrated exceptionally reduced virulence. The particularly mutated strain *F. graminearum* negatively affected the pathogenicity and conidia formation of the fungi [59]. When functionally analyzed, the TOP1 gene from the Basidiomycete plant pathogen *Ustilago maydis* demonstrated that vegetative growth did not require it, but a pigmentation defect was exhibited by its mutant [60]. From this information, our study revealed that Topoisomerase 1 was necessary for RS15 virulence. The proteins P0CM04 and P0CM05 were linked with actin-like protein ARP6, which were overexpressed in the virulent isolate. Actin is one of the most fundamental and abundant proteins in eukaryotic cells. ARP6, an actin-related protein, is essential for nucleolar function and structure. Further, it has a vital role in transcriptional regulation and DNA repair [61,62]. This finding supports that actin-like protein ARP6 up-regulation in the virulent isolate was vital for essential metabolism and transcriptional activities. Squalene synthase (Q92459) was over-expressed in the virulent isolate RS15. Squalene is a polyunsaturated terpene, an intermediate in the ergosterol biosynthetic pathway, playing a significant role in fungal cell structure. Squalene levels influence ergosterol biosynthesis [63]. Primarily, the squalene synthase Erg9 enzyme used two molecules of farnesyl-PP to form squalene, the precursor of all steroids. All eukaryotic organisms have essential molecules called sterols, and various genetic mutations that eliminate the enzymatic steps in sterol biosynthesis were proven to be lethal [64]. Numerous antifungal agents, including azoles, target the sterol biosynthesis pathway, which results in the production of ergosterol, a significant component of the fungal plasma membrane [65]. The secondary metabolite compounds ergosterol and their biological function were identified from *R. solani* [66,67,68]. Moreover, overexpressed squalene synthase enzyme in virulent isolate may promote the proper function of critical molecular events, particularly in ergosterol synthesis.

The protein A8NV38, cyanate hydratase, is over-expressed in virulent isolate, which plays a vital role in nitrogen assimilation or cyanate detoxification in bacteria, fungi and plants [69,70,71]. Cyanate, an important cyanide derivative, is formed by the oxidation of a toxic substance, cyanide. The whole-genome sequencing and secretome analysis of *Thermomyces lanuginosus* demonstrated the presence of cyanase (Tl-Cyn), and the overexpressed Tl-Cyn gene was evaluated for cyanate detoxification activity [72,73,74,75,76]. In our study, the virulent *R. solani* had overexpressed cyanases and hypothesized that virulent *R. solani* could tolerate plant-produced and another microbial cyanate in the soil environment. The protein Q6QWR1 is linked with pyranose 2-oxidase (Pox2), a flavin-dependent oxidoreductase [77]. It oxidizes D-glucose as well as other monosaccharide substrates of hydrogen peroxide. Pox is typically found in basidiomycetes fungi, where it is associated extracellularly with membrane-bound vesicles or other membrane structures in the periplasmic space of hyphae [78,79]. The hydrogen peroxide (H_2_O_2_) secreted by the fruiting bodies of *Tricholoma matsutake*, produced by pyranose oxidase, strongly inhibited mycelial growth of the phytopathological fungus *R. solani*. This data suggested a high-level expression of pyranose oxidase that plays a significant role in the antifungal activity, thereby dominating the microbial communities [80]. The role of some up-regulated proteins, such as Protein EFR, Pre-mRNA-splicing factor, Lon protease homolog, O-acetyltransferase, rRNA biogenesis protein, mRNA cleavage and polyadenylation factor CLP1 on the virulence or pathogenicity of *R. solani,* is unknown.

A total of 21 proteins were under-expressed in RS15 when compared to RS22. For instance, Elongation factor G (Q4P257), Sulfate adenylyltransferase (Q4P460), 5′-3′ exoribonuclease 2(Q4P149), Glyceraldehyde-3-phosphate dehydrogenase 1 (P32635), Inositol-pentakisphosphate 2-kinase (Q4P4C1) and Pentafunctional AROM polypeptide (P0CM22 and P0CM23) were responsible for translation, biosynthesis of sulfur-containing amino acids, protein biosynthesis, glycolysis, phosphorylation and catalytic activities in fungi, respectively [81,82,83,84]. These proteins were hypothesized to be essential for fungal cellular and metabolism rather than virulence-related activities.

## 5. Conclusions

A total of 58 *R. solani* isolates were obtained from rice-growing areas of Odisha, Eastern India. They displayed differences in virulence on the rice cultivar, Tapaswini. The isolate RS15 was found to be the most virulent and RS22 was identified as the least virulent. The virulence of RS15 and RS22 was further confirmed on other rice genotypes viz., Pusa Basmati-1, Vanaprabha, Hazaridhan, Swarna, Lunishree, Savitri, Sadabahar and TN1. Further, we employed an LC-MS/MS-based proteomic approach to profile the proteomic differences between the virulent RS15 isolate and less virulent RS22 isolate. A total of 48 differentially expressed proteins were identified, of which 27 proteins were up-regulated in virulent isolate. These up-regulated proteins were more responsible for pathogenicity, detoxification, antifungal activity, sporulation, fungal development, morphology, essential metabolism and transcriptional activities. The important proteins, such as sorting nexin-4, JmjC domain-containing histone demethylation protein 1, topoisomerase 1, squalene synthase, pyranose 2-oxidase, Hsp70 and cyanate hydratase, may have major roles in the pathogenicity and virulence of *R. solani* RS15. The proteomic information generated in this study contributes knowledge on the virulence mechanism responsible for the sheath blight of rice disease. Further research is required to explore the importance of these proteins and their possible roles during sheath blight disease establishment.

## Figures and Tables

**Table 1:** Pathogenicity of *R. solani* isolates on Tapaswini.

Isolates	PDI *	Average Virulent Index	Disease Reaction
RS1	25.93 (30.61) ^k^	2.33	Less virulent
RS2	25.93 (30.61) ^k^	2.33	Less virulent
RS3	33.33 (35.26) ^j^	3.00	Less virulent
RS4	25.93 (30.61) ^k^	2.33	Less virulent
RS5	33.33 (35.26) ^j^	3.00	Less virulent
RS6	18.52 (25.49) ^l^	1.67	Less virulent
RS7	25.93 (30.61) ^k^	2.33	Less virulent
RS8	18.52 (25.49) ^l^	1.67	Less virulent
RS9	77.78 (61.93) ^d^	7.00	Moderately virulent
RS10	77.78 (61.93) ^d^	7.00	Moderately virulent
RS11	85.19 (67.51) ^c^	7.67	Moderately virulent
RS12	48.15 (43.94) ^h^	4.33	Moderately virulent
RS13	33.33 (35.26) ^j^	3.00	Less virulent
RS14	77.78 (61.93) ^d^	7.00	Moderately virulent
RS15	100.00 (88.19) ^a^	9.00	Highly virulent
RS16	11.11 (19.47) ^m^	1.00	Less virulent
RS17	11.11 (19.47) ^m^	1.00	Less virulent
RS18	55.56 (48.20) ^g^	5.00	Moderately virulent
RS19	33.33 (35.26) ^j^	3.00	Less virulent
RS20	18.52 (25.49) ^l^	1.67	Less virulent
RS21	18.52 (25.49) ^l^	1.67	Less virulent
RS22	11.11 (19.47) ^m^	1.00	Less virulent
RS23	77.78 (61.93) ^d^	7.00	Moderately virulent
RS24	40.74 (39.66) ^i^	3.67	Less virulent
RS25	40.74 (39.66) ^i^	3.67	Less virulent
RS26	11.11 (19.47) ^m^	1.00	Less virulent
RS27	11.11 (19.47) ^m^	1.00	Less virulent
RS28	77.78 (61.93) ^d^	7.00	Moderately virulent
RS29	25.93 (30.61) ^k^	2.33	Less virulent
RS30	25.93 (30.61) ^k^	2.33	Less virulent
RS31	55.56 (48.20) ^g^	5.00	Moderately virulent
RS32	48.15 (43.94) ^h^	4.33	Moderately virulent
RS33	18.52 (25.49) ^l^	1.67	Less virulent
RS34	92.59 (74.77) ^b^	8.33	Highly virulent
RS35	25.93 (30.61) ^k^	2.33	Less virulent
RS36	25.93 (30.61) ^k^	2.33	Less virulent
RS37	70.37 (57.05) ^e^	6.33	Moderately virulent
RS38	33.33 (35.26) ^j^	3.00	Less virulent
RS39	33.33 (35.26) ^j^	3.00	Less virulent
RS40	33.33 (35.26) ^j^	3.00	Less virulent
RS41	33.33 (35.26) ^j^	3.00	Less virulent
RS42	33.33 (35.26) ^j^	3.00	Less virulent
RS43	33.33 (35.26) ^j^	3.00	Less virulent
RS44	62.96 (52.52) ^f^	5.67	Moderately virulent
RS45	62.96 (52.52) ^f^	5.67	Moderately virulent
RS46	33.33 (35.26) ^j^	3.00	Less virulent
RS47	40.74 (39.66) ^i^	3.67	Less virulent
RS48	55.56 (48.20) ^g^	5.00	Moderately virulent
RS49	77.78 (61.93) ^d^	7.00	Moderately virulent
RS50	33.33 (35.26) ^j^	3.00	Less virulent
RS51	11.11 (19.47) ^m^	1.00	Less virulent
RS52	11.11 (19.47) ^m^	1.00	Less virulent
RS53	11.11 (19.47) ^m^	1.00	Less virulent
RS54	18.52 (25.49) ^l^	1.67	Less virulent
RS55	48.15 (43.94) ^h^	4.33	Moderately virulent
RS56	40.74 (39.66) ^i^	3.67	Less virulent
RS57	11.11 (19.47) ^m^	1.00	Less virulent
RS58	11.11 (19.47) ^m^	1.00	Less virulent

* PDI recorded on 28th days after pathogen inoculation. Values are the mean of three replications. Values in the parenthesis are arcsine-transformed values. Means in a column followed by same superscript letter are not significantly different at the 5% level by DMRT.

**Table 2:** List of differentially expressed proteins among virulent (RS15) and less virulent (RS22) isolates.

Accessions	Description	Fold Change **	Category
P0CN36	Protein EFR3	2.034	Up-regulated
Q4P3U5	Protein EFR3	2.034	Up-regulated
* P10248	Phenylalanine ammonia-lyase	2.054	Up-regulated
Q4PB37	Pre-mRNA-splicing factor	2.181	Up-regulated
Q4P0P3	Mediator of RNA polymerase II transcription subunit 14	2.270	Up-regulated
P0CQ16	Lon protease homolog, mitochondrial	2.293	Up-regulated
Q6QWR1	Pyranose 2-oxidase	2.411	Up-regulated
* Q92459	Squalene synthase	2.664	Up-regulated
Q4P652	Pre-mRNA-splicing factor CEF1	2.691	Up-regulated
* A8NV38	Cyanate hydratase	2.718	Up-regulated
P0CM56	Probable O-acetyltransferase CAS1	2.773	Up-regulated
Q4P9P3	ATP-dependent RNA helicase DRS1	2.829	Up-regulated
P32186	Elongation factor 1-alpha	3.004	Up-regulated
P0CR93	Topoisomerase 1-associated factor 1	3.034	Up-regulated
P0CM04	Actin-like protein ARP6	3.096	Up-regulated
* P0CM05	Actin-like protein ARP6	3.706	Up-regulated
Q4P3H6	Actin cytoskeleton-regulatory complex protein SLA1	4.904	Up-regulated
* P0CR92	Topoisomerase 1-associated factor 1	5.207	Up-regulated
A8Q513	Exportin-T	8.935	Up-regulated
Q4P525	Glutamyl-tRNA (Gln) amidotransferase subunit B, mitochondrial	8.935	Up-regulated
A8N142	rRNA biogenesis protein RRP36	9.116	Up-regulated
P18694	Heat shock 70 kDa protein 2	15.800	Up-regulated
* P0CO40	JmjC domain-containing histone demethylation protein 1	17.385	Up-regulated
A8PTG4	tRNA-dihydrouridine(47) synthase [NAD(P)(+)]	3.265	Up-regulated
P0CO41	JmjC domain-containing histone demethylation protein 1	14.604	Up-regulated
A8PWG8	mRNA cleavage and polyadenylation factor CLP1	2.836	Up-regulated
P0CR62	Sorting nexin-4	17.915	Up-regulated
* Q4P763	GMP synthase [glutamine-hydrolyzing]	0.018	Down-regulated
Q4P257	Elongation factor G, mitochondrial	0.024	Down-regulated
* P34809	Glycylpeptide N-tetradecanoyltransferase	0.072	Down-regulated
* Q4P460	Sulfate adenylyltransferase	0.124	Down-regulated
Q4P149	5′-3′ exoribonuclease 2	0.157	Down-regulated
* P32635	Glyceraldehyde-3-phosphate dehydrogenase 1	0.160	Down-regulated
P0CO63	Protein phosphatase methylesterase 1	0.247	Down-regulated
P0CM63	Protein CFT1	0.259	Down-regulated
P0CM89	Endoribonuclease	0.267	Down-regulated
P0CM88	Endoribonuclease	0.292	Down-regulated
P0CM62	Protein CFT1	0.292	Down-regulated
P0CO62	Protein phosphatase methylesterase 1	0.298	Down-regulated
Q4P4C1	Inositol-pentakisphosphate 2-kinase	0.307	Down-regulated
P0CO60	Putative lipase ATG15	0.333	Down-regulated
P0CM23	Pentafunctional AROM polypeptide	0.343	Down-regulated
P0CO61	Putative lipase ATG15	0.353	Down-regulated
P0CP58	Pescadillo homolog	0.427	Down-regulated
Q4P0P0	Eukaryotic translation initiation factor 3 subunit C	0.440	Down-regulated
I3ZNU9	Orsellinic acid synthase ArmB	0.458	Down-regulated
A0A1B1ZGB5	Adenylate-forming reductase Nps10	0.458	Down-regulated
* P0CM22	Pentafunctional AROM polypeptide	0.487	Down-regulated

* Proteins selected for qPCR analysis. ** Fold change >2 is considered for up-regulated and <0.5 is down-regulated.

## Data Availability

The data presented in this study are available on request from the corresponding author.

## Supplementary Materials

The following supporting information can be downloaded at: https://www.mdpi.com/article/10.3390/jof8040370/s1, Table S1: List of gene-specific primers for qRT-PCR.

## References

[1] Dolfors F., Holmquist L., Dixelius C., Tzelepis G. (2019). A LysM effector protein from the basidiomycete *Rhizoctonia solani* contributes to virulence through suppression of chitin triggered immunity. Mol. Genet. Genom..

[2] Bernardes-De-Assis J., Storari M., Zala M., Wang W., Jiang D., Shidong L., Jin M., McDonald B.A., Ceresini P.C. (2009). Genetic structure of populations of the rice-infecting pathogen *Rhizoctonia solani* AG 1 IA from china. Phytopathology.

[3] Kumar K.V.K., Raju S.K., Reddy M.S., Kloepper J.W., Lawrence K.S., Groth D.E., Miller M.E., Sudini H., Du B.H. (2009). Evaluation of commercially available PGPR for control of rice sheath blight caused by *Rhizoctonia solani*. J. Pure Appl. Microbiol..

[4] Ghosh S., Gupta S.K., Jha G. (2014). Identification and functional analysis of AG1-IA specific genes of *Rhizoctonia solani*. Curr. Genet..

[5] Taheri P.H.M. (2007). Riboflavin-induced resistance against rice sheath blight functions through the potentiation of lignin formation and jasmonic acid signalling pathway. Commun. Agric. Appl. Biol. Sci..

[6] Ou S.H. (1985). Rice Diseases.

[7] Rauf C., Ahmad I., Ashraf M. (2007). Anastomosis groups of *Rhizoctonia solani* Kuhn isolates from potato in Pakistan. Pak. J. Bot..

[8] Sharon M., Sneh B., Kuninaga S., Hyakumachi M., Naito S. (2008). Classification of *Rhizoctonia* spp. using rDNA-ITS sequence analysis supports the genetic basis of the classical anastomosis grouping. Mycoscience.

[9] Wang X., Jiang N., Liu J., Liu W., Wang G.L. (2014). The role of effectors and host immunity in plant-necrotrophic fungal interactions. Virulence.

[10] Zheng A., Lin R., Zhang D., Qin P., Xu L., Ai P., Ding L., Wang Y., Chen Y., Liu Y. (2013). The evolution and pathogenic mechanisms of the rice sheath blight pathogen. Nat. Commun..

[11] Nagarajkumar M., Jayaraj J., Muthukrishnan S., Bhaskaran R., Velazhahan R. (2005). Detoxification of oxalic acid by *Pseudomonas fluorescens* strain PfMDU2: Implications for the biological control of rice sheath blight caused by *Rhizoctonia solani*. Microbiol. Res..

[12] Rao T.B., Chopperla R., Methre R., Punniakotti E., Venkatesh V., Sailaja B., Sundaram R.M. (2019). Pectin induced transcriptome of a *Rhizoctonia solani* strain causing sheath blight disease in rice reveals insights on key genes and RNAi machinery for development of pathogen derived resistance. Plant Mol. Biol..

[13] Lore J.S., Jain J., Hunjan M.S., Gargas G., Mangat G.S., Sandhu J.S. (2015). Virulence spectrum and genetic structure of *Rhizoctonia* isolates associated with rice sheath blight in the northern region of India. Eur. J. Plant Pathol..

[14] Kim B.S., Hwang B.K. (2007). Microbial fungicides in the control of plant diseases. J. Phytopathol..

[15] Yang Y., Zhang H., Li G., Li W., Wang X., Song F. (2009). Ectopic expression of MgSM1, a Cerato-platanin family protein from *Magnaporthe grisea*, confers broad-spectrum disease resistance in Arabidopsis. Plant Biotechnol. J..

[16] Berg G. (2009). Plant-microbe interactions promoting plant growth and health: Perspectives for controlled use of microorganisms in agriculture. Appl. Microbiol. Biotechnol..

[17] Komatsu S., Tanaka N. (2005). Rice proteome analysis: A step toward functional analysis of the rice genome. Proteomics.

[18] Wang Y., Wu J., Park Z.Y., Kim S.G., Rakwal R., Agrawal G.K. (2011). Comparative secretome investigation of *Magnaporthe oryzae* proteins responsive to nitrogen starvation. J. Proteome Res..

[19] Prabhukarthikeyan S.R., Manikandan R., Durgadevi D., Keerthana U., Harish S., Karthikeyan G., Raguchander T. (2017). Bio-suppression of turmeric rhizome rot disease and understanding the molecular basis of tripartite interaction among *Curcuma longa*, *Pythium aphanidermatum* and *Pseudomonas fluorescens*. Biol. Control.

[20] Manikandan R., Harish S., Karthikeyan G., Raguchander T. (2018). Comparative proteomic analysis of different isolates of *Fusarium oxysporum* f.sp. *lycopersici* to exploit the differentially expressed proteins responsible for virulence on tomato plants. Front. Microbiol..

[21] Prabhukarthikeyan S.R., Yadav M.K., Anandan A., Aravindan S., Keerthana U., Raghu S., Baite M.S., Parameswaran M.S., Panneerselvam M.S., Rath P.C. (2019). Bio-protection of brown spot disease of rice and insight into the molecular basis of interaction between *Oryza sativa*, *Bipolaris oryzae* and *Bacillus amyloliquefaciens*. Biol. Control.

[22] Xu S., Chen J., Liu L., Wang X. (2007). Proteomics associated with virulence differentiation of *Curvularia lunata* in Maize in China. J. Integr. Plant Biol..

[23] Kwon Y.S., Kim S.G., Chung W.S., Bae H., Jeong S.W., Shin S.C., Jeong M.J., Park S.C., Kwak Y.S., Bae D.W. (2014). Proteomic analysis of *Rhizoctonia solani* AG-1 sclerotia maturation. Fungal Biol..

[24] Anderson J.P., Sperschneider J., Win J., Kidd B., Yoshida K., Hane J., Diane G.O., Saunders D.G.O., Singh K.B. (2017). Comparative secretome analysis of *Rhizoctonia solani* isolates with different host ranges reveals unique secretomes and cell death inducing effectors. Sci. Rep..

[25] Lakshman D., Roberts D.P., Garrett W.M., Natarajan S.S., Omar D., Alkharouf N., Pain A., Khan F., Jambhulkar P.P., Mitra A. (2016). Proteomic Investigation of *Rhizoctonia solani* AG 4 Identifies Secretome and Mycelial Proteins with roles in Plant Cell Wall Degradation and Virulence. J. Agric. Food Chem..

[26] Anderson J.P., Hane J.K., Stoll T., Pain N., Hastie M.L., Kaur P., Hoogland C., Gorman J.J., Singh K.B. (2016). Proteomic Analysis of *Rhizoctonia solani* Identifies Infection-specific, Redox Associated Proteins and Insight into Adaptation to Different Plant Hosts. Mol. Cell. Proteom..

[27] Ma H., Sheng C., Qiao C., Zhao H., Niu D. (2019). A comparative proteomic approach to identify defence-related proteins between resistant and susceptible rice cultivars challenged with the fungal pathogen *Rhizoctonia solani*. Plant Growth Regul..

[28] Mora L., Bramley P.M., Fraser P.D. (2013). Development and optimisation of a label-free quantitative proteomic procedure and its application in the assessment of genetically modified tomato fruit. Proteomics.

[29] Li L., Luo Z., Huang X., Zhang L., Zhao P., Ma H., Li X., Ban Z., Liu X. (2015). Label-free quantitative proteomics to investigate strawberry fruit proteome changes under controlled atmosphere and low temperature storage. J. Proteom..

[30] Sharma N.R., Teng P.S., Oliver F.M. (1990). Comparisons of assessment methods for rice sheath blight disease. Philipp. Phytopathol..

[31] IRRI (2013). Standard Evaluation System (SES) of Rice (Revised).

[32] Prasad B., Eizenga G.C. (2008). Rice sheath blight disease resistance identified in *Oryza* species accessions. Plant Dis..

[33] Wheeler B.E.J. (1969). An Introduction to Plant Diseases.

[34] White T.J., Bruns T., Lee S., Taylor J. (1990). Amplification and direct sequencing of fungalribosomal RNA genes for phylogenetics. PCR Protocols. A Guide to Methods and Applications.

[35] Matsumoto M. (2002). Trials of direct detection and identification of *Rhizoctonia solani* AG1 and AG 2 subgroups using specifically primed PCR analysis. Mycoscience.

[36] Meng H., Wang S., Yang W., Ding X., Li N., Chu Z., Li X. (2021). Identification of virulence associated milRNAs and their bidirectional targets in *Rhizoctonia solani* and maize during infection. BMC Plant Biol..

[37] Gomez K.A., Gomez A.A. (1984). Statistical Procedure for Agricultural Research.

[38] Perez-Riverol Y., Csordas, Bai A., Bernal-Llinares J., Hewapathirana M., Kundu S., Inuganti D.J., Griss A., Mayer J., Eisenacher G. (2019). The PRIDE database and related tools and resources in 2019: Improving support for quantification data. Nucleic Acids Res..

[39] Deutsch E.W., Bandeira N., Sharma V., Perez-Riverol Y., Carver J.J., Kundu D.J., Garcia-Seisdedos D., Jarnuczak A.F., Hewapathirana S., Pullman B.S. (2020). The ProteomeXchange consortium in 2020: Enabling ‘big data’ approaches in proteomics. Nucleic Acids Res..

[40] Adhipathi P., Singh V., Meena S.C. (2013). Virulence diversity of *Rhizoctonia solani* causing sheath blight disease in rice and its host pathogen interaction. Bioscan.

[41] Singh R., Murti S., Mehilalm T.A., Prasad D. (2015). Virulence Diversity in *Rhizoctonia solani* Causing Sheath Blight in Rice. J. Plant Pathol. Microb..

[42] Pavani S.L., Singh V. (2018). Assessment of virulence diversity of *Rhizoctonia solani* causing sheath blight disease in rice from Eastern Up. Curr. J. Appl. Sci. Technol..

[43] El-Shafey R.A.S., Elamawi R.M., Saleh M.M., Tahoon A.M., Emeran A.A. (2019). Morphological, pathological and molecular characterisation of rice sheath blight disease causal organism *Rhizoctonia solani* AG-1 IA in Egypt. Arch. Phytopathol. Plant Prot..

[44] Yu Y., Chun-Hao J., Chao W., Liu-Jun C., Li H., Xu Q., Jian-Hua G. (2017). An improved strategy for stable biocontrol agents selecting to control rice sheath blight caused by *Rhizoctonia solani*. Microbiol. Res..

[45] Shu C., Zou C., Chen J.L., Fang T., Run H.Y., Er-Xun Z. (2014). Genetic diversity and population structure of *Rhizoctonia solani* AG-1 IA, the causal agent of rice sheath blight, in South China. Can. J. Plant Pathol..

[46] Moni Z.R., Ali M.A., Alam M.S., Rahman M.A., Bhuiyan M.R., Mian M.S., Iftekharuddaula K.M., Latif M.A., Khan M.A.I. (2016). Morphological and Genetical Variability among *Rhizoctonia solani* isolates causing sheath blight disease of rice. Rice Sci..

[47] Nagaraj B.T., Sunkad G., Pramesh D., Manjunath K.N., Patil M.B., Yadav M.K., Patil N.B. (2019). Morphological, genetic and virulence diversity of *Rhizoctonia solani* isolates from different rice growing regions of Southern India. Res. J. Biotechnol..

[48] Deng Y.Z., Qu Z., He Y., Naqvi N.I. (2012). Sorting nexinSnx41 is essential for conidiation and mediates glutathione-based antioxidant defense during invasive growth in *Magnaporthe oryzae*. Autophagy.

[49] Zheng H., Guo Z., Xi Y., Yuan M., Lin Y., Wu C., Abubakar Y.S., Dou X., Li G., Wang Z. (2017). Sorting nexin (MoVps17) is required for fungal development and plant infection by regulating endosome dynamics in the rice blast fungus. Environ. Microbiol..

[50] Gacek-Matthews A., Berger H., Sasaki T., Wittstein T., Gruber C., Lewis Z.A., Strauss J. (2016). KdmB, a Jumonji Histone H3 Demethylase, Regulates Genome-Wide H3K4 Trimethylation and Is Required for Normal Induction of Secondary Metabolism in *Aspergillus nidulans*. PLoS Genet..

[51] Bachleitner S., Sorensen J.L., Gacek-Matthews A., Sulyok M., Studt L., Strauss J. (2019). Evidence of a Demethylase-Independent Role for the H3K4-Specific Histone Demethylases in *Aspergillus nidulans* and *Fusarium graminearum* Secondary Metabolism. Front. Microbiol..

[52] Huh A., Dubey A., Kim S., Jeon J., Lee Y. (2017). *MoJMJ1*, encoding a histone demethylase containing JmjC domain, is required for pathogenic development of the rice blast fungus, *Magnaporthe oryzae*. Plant Pathol. J..

[53] Li Z., Srivastava P. (2004). Heat-shock proteins. Curr. Protoc. Immunol..

[54] Chen L., Geng X., Ma Y., Zhao J., Che W., Xing X., Shi Y., Sun B., Li H. (2019). The ER Lumenal Hsp70 Protein FpLhs1 Is Important for Conidiation and Plant Infection in *Fusarium pseudograminearum*. Front. Microbiol..

[55] Presti L.L., LopezDiaz C., Turra D., di Pietro A., Hampel M., Heimel K., Kahmann R. (2016). A conserved co-chaperone is required for virulence in fungal plant pathogens. New Phytol..

[56] Yang J., Liu M., Liu X., Yin Z., Sun Y., Zhang H., Zheng X., Wang P., Zhang Z. (2018). Heat shock proteins MoSsb1, MoSsz1, and MoZuo1 attenuate MoMkk1-mediated CWI signaling and are important for growth and pathogenicity of *Magnaporthe oryzae*. Mol. Plant-Microbe Interact..

[57] Bui D., Lee Y., Lim J.Y., Fu M., Kim J., Choi G.J., Son H., Lee Y.W. (2016). Heat shock protein 90 is required for sexual and asexual development, virulence, and heat shock response in *Fusarium graminearum*. Sci. Rep..

[58] Wang J.C. (2002). Cellular roles of DNA topoisomerases: A molecular perspective. Nat. Rev. Mol. Cell Biol..

[59] Baldwin T.K., Urban M., Brown N., Hammond-Kosack K.E. (2010). A role for topoisomerase in *Fusarium graminearum* and *F. culmorum* pathogenesis and sporulation. Mol. Plant Microbe Interact..

[60] Gerhold D., Thiyagarajan M., Kmiec E.B. (1994). The topoisomerase I gene from *Ustilago maydis*: Sequence, disruption and mutant phenotype. Nucleic Acids Res..

[61] Klages-Mundt N.L., Kumar A., Zhang Y., Kapoor P., Shen X. (2018). The nature of actin family proteins in chromatin-modifying complexes. Front. Genet..

[62] Kitamura H., Matsumori H., Kalendova A., Hozak P., Goldberg I.G., Nakao M., Saitoh N., Harata M. (2015). The actin family protein ARP6 contributes to the structure and the function of the nucleolus. Biochem. Biophys. Res. Commun..

[63] Garaiova M., Zambojova V., Simova Z., Griac P., Hapala I. (2013). Squalene epoxidase as a target for manipulation of squalene levels in the yeast *Saccharomyces cerevisiae*. FEMS Yeast Res..

[64] Karst F., Lacroute F. (1977). Ergosterol biosynthesis in *Saccharomyces cerevisiae*: Mutants deficient in the early steps of the pathway. Mol. Gen. Genet..

[65] Zhang Y., Yang H., Turra D., Zhou S., HazalAyhan D., DeIulio G.A., Guo L., Broz K., Wiederhold N., Coleman J.J. (2020). The genome of opportunistic fungal pathogen *Fusarium oxysporum* carries a unique set of lineage-specific chromosomes. Commun. Biol..

[66] Ma Y.M., Li Y., Liu J.Y., Song Y.C., Tan R.X. (2004). Anti-*Helicobacter pylori* metabolites from *Rhizoctonia* sp. Cy064, an endophytic fungus in *Cynodondactylon*. Fitoterapia.

[67] Aliferis K.A., Jabaji S. (2010). 1H NMR and GC-MS metabolic fingerprinting of developmental stages of *Rhizoctonia solani* sclerotia. Metabolomics.

[68] Liang X., Wang X.-H., Luo R.-Y., Lu S.-Q., Guo Z.-J., Wang M.-A., Liu Y., Zhou L.-G. (2015). Secondary metabolites of rice sheath blight pathogen *Rhizoctonia solani* Kuhn and their biological activities. J. Integr. Agric..

[69] Elleuche S., Poggeler S.A. (2008). Cyanase is transcriptionally regulated by arginine and involved in cyanate decomposition in *Sordaria macrospora*. Fungal Genet. Biol..

[70] Marsalova L., Vitamvas P., Hynek R., Prasil I.T., Kosova K. (2016). Proteomic Response of *Hordeum vulgare* cv. Tadmor and *Hordeum marinum* to Salinity Stress: Similarities and Differences between a Glycophyte and a Halophyte. Front. Plant Sci..

[71] Linder T. (2019). Cyanase-independent utilization of cyanate as a nitrogen source in ascomycete yeasts. World J. Microb. Biot..

[72] Mchunu N.P., Permaul K., Abdul Rahman A.Y., Saito J.A., Singh S., Alam M. (2013). Xylanase Super producer: Genome sequence of a compost-loving thermophilic fungus, *Thermomyces lanuginosus* Strain SSBP. Genome Announc..

[73] Winger A.M., Heazlewood J.L., Chan L.J.G., Petzold C.J., Permaul K., Singh S. (2014). Secretome analysis of the thermophilic xylanase hyper-producer *Thermomyces lanuginosus* SSBP cultivated on corn cobs. J. Ind. Microbiol. Biotechnol..

[74] Ranjan B., Pillai S., Permaul K., Singh S. (2017). Expression of a novel recombinant cyanate hydratase (rTl-Cyn) in *Pichia pastoris*, characteristics and applicability in the detoxification of cyanate. Bioresour. Technol..

[75] Ranjan B., Pillai S., Permaul K., Singh S. (2018). A novel strategy for the efficient removal of toxic cyanate by the combinatorial use of recombinant enzymes immobilized on aminosilane modified magnetic nanoparticles. Bioresour. Technol..

[76] Ranjan B., Pillai S., Singh K.P.S. (2019). Simultaneous removal of heavy metals and cyanate in a wastewater sample using immobilized cyanate hydratase on magnetic-multiwall carbon nanotubes. J. Hazard. Mater..

[77] Sutzl L., Laurent C.V.F.P., Abrera A.T., Schutz G., Ludwig R., Haltrich D. (2018). Multiplicity of enzymatic functions in the CAZy AA3 family. Appl. Microbiol. Biotechnol..

[78] Daniel G., Volc J., Kubatova E., Nilsson T. (1992). Ultrastructural and immunocytochemical studies on the H_2_O_2_-producing enzyme pyranose oxidase in *Phanerochaete chrysosporium* grown under liquid culture conditions. Appl. Environ. Microbiol..

[79] Abrera A.T., Sutzl L., Haltrich D. (2020). Pyranose oxidase: A versatile sugar oxidoreductase for bioelectrochemical applications. Bioelectrochemistry.

[80] Takakura Y. (2015). *Tricholoma matsutake* fruit bodies secrete hydrogen peroxide as a potent inhibitor of fungal growth. Can. J. Microbiol..

[81] Marzluf G.A. (1997). Molecular genetics of sulfur assimilation in filamentous fungi and yeast. Annu. Rev. Microbiol..

[82] Lima J.O., Pereira J.F., Rincones J., Barau J.G., Araujo E.F., Pereira G.A.G., Queiroz M.V. (2009). The glyceraldehyde-3-phosphate dehydrogenase gene of Moniliophthora perniciosa, the causal agent of witches’ broom disease of The obroma cacao Genet. Mol. Biol..

[83] Miki T.S., Grobhans H. (2013). The multifunctional RNase XRN2. Biochem. Soc. Trans..

[84] Mateyak M.K., Pupek J.K., Garino A.E., Knapp M.C., Colmer S.F., Kinzy T.G., Dunaway S. (2018). Demonstration of translation elongation factor 3 activity from a non-fungal species, *Phytophthora infestans*. PLoS ONE.

