# Supplementary material for: Comparative Proteomic Analysis of Rhizoctonia solani Isolates Identifies the Differentially Expressed Proteins with Roles in Virulence
Source: J Fungi (Basel). 2022 Apr 5;8(4):370. doi: 10.3390/jof8040370 (PMC9029756; doi:10.3390/jof8040370)
Supplement: Supplementary file 1 [file jof-08-00370-s001.zip › jof-1643640-supplementary.pdf]

Table S1: List of gene-specific primers for qRT-PCR

Genes	Forward	Reverse
Phenylalanine ammonia-lyase	CCAATACTAATCCCGAAGGTCTC	GACCAAACGCATAATCTCCATTC
Squalene synthase	GAACTCGGACTTCCCGAAA	CGCGCATACAAGCAGAAAG
Cyanate hydratase	GGGTCTTGGTGTATGGGTATC	GTTTCCTGTCCACCGTTACA
Actin-like protein ARP6	GGATGTTCAATGGTGGTGGA	CATTTGCCGCTGACAAGTTATT
Topoisomerase 1-associated factor 1	ACCTGAGGAGAGTCCAAAGA	ACTGGAATTCGTGGACGTG
JmjC domain histone demethylation protein 1	GACAAGGACCATGATGGGAAA	ATGGGCACATCCACATCTTT
GMP synthase (glutamine-hydrolizing)	ATCTGCTCCCATATTCGAAGTC	GGCGCAAGGATTTCATGTTC
Glycylpeptide N-tetradecanoyltransferase	CAATGCTCTCACCCTCATGG	CGGTTCGCCAGTTGTAAAGA
Sulfate adenylyltransferase	TGCTGGCGGAAACTTCTT	TCATCGACACCAGTGAAACC
Glyceraldehyde-3-phosphate dehydrogenase	GTTGGCGATGAACACTCTTCTA	ATAGGCGATCAGGTCAACAAC
Pentafunctional AROM polypeptide	CCGGAGATGCTTCAGTTGAT	TCCTTCCCTACCACGACTAC
